# Western Alliance 5^th^ Annual Symposium, in collaboration with the National Centre for Farmer Health, Hamilton, Victoria, Australia 12 –14^th^ Sept 2018

**DOI:** 10.1097/MD.0000000000017909

**Published:** 2019-12-10

**‘Toward a healthy rural and regional Australia’:***Hamilton, Victoria, Australia 12th–14th Sept, 2018.*

In meeting the challenge of an existing health-gap between rural and metropolitan Australians, the 5th Annual Symposium of Western Alliance https://www.westernalliance.org.au/ was held in collaboration with Western District Health Service https://www.wdhs.net/ and the National Centre for Farmer Health https://www.farmerhealth.org.au/. This symposium brought together researchers, educators, policy makers and health professionals to share research knowledge, calibrate motivations and to translate local and international research into best clinical practice. The theme of the conference acknowledges an enduring challenge of our healthcare providers and academic partners with collective responsibility to maintain a regional/rural focus in coalition of shared efforts to promoting evidence based research practices towards optimal patient care.

Incoming Executive Director, Professor Warren Payne, in collaboration with the scientific advisory and leadership team (SaLT) and organizing committee extended gratitude to sponsors, exhibitors, delegates and to symposium conveyor Dr Renée Otmar for efforts in support of a successful 5^th^ Annual Symposium of the Western Alliance. Professor Brendan Crotty, Interim Chair of the Western Alliance Board, closed proceedings by highlighting some remarkable accomplishments of Professor Susan Brumby *and colleagues*, in celebrating the 10th anniversary of the National Centre for Farmer Health.

Symposium abstracts shared herein are showcased to demonstrate a sample of research success during 2018 and to further promote a united agenda to promote research informed clinical practice in regional and rural Victoria.

**Mr Drew Aras**^**1**^**, Executive Officer**

^*1*^*Western Alliance Health Research Ltd, 2, Myers House, PO Box 281, Geelong VIC 3220 AUSTRALIA*

## 1 SYMPOSIUM ABSTRACTS

**S01 - Rural policy makers’ views of the evidence for heart disease prevention policy**

Alston L, Nichols M, Allender S.

Global Obesity Centre, Deakin University, Australia

**Aim:** To describe the barriers and enablers to the use of scientific evidence in rural health policy decisions in the rural context. To compare views between local, and state and federal government participants.

**Methods:** Policy makers and government advisers who worked on rural health policy at local, state and federal government levels, with specific focus on the state of Victoria (n = 9) participated in semi-structured qualitative interviews (n = 21). Thematic analysis was undertaken applying two theoretical perspectives: context-based evidence-based policy making and the conceptual framework for understanding rural and remote health.

**Results:** Social culture and low resourcing within the rural context were seen as limiting the potential for evidence based policy making at local government level in rural Victoria. Differing levels of political pressures within the broader health system was also seen as a constraints for adoption and development of evidence- based policy in rural communities. Participants described potential for policy to have a greater impact on reducing heart disease in rural areas. Scientific studies were less valued as evidence than local anecdote stories to prioritise specific policy.

**Conclusion:** The rural context constrains the use of scientific evidence in policy making for the prevention of heart disease in rural areas. There appears to be a missed opportunity for evidence based chronic disease prevention activities within rural local governments in Victoria.

Presenting author: Laura Alston laura.alston@deakin.edu.au

**S02 - Assessing community readiness for a mental health promotion program in rural Tasmania**

Auckland SR, Bridgman H, Mond J, Kent K, Hoang H, De Deuge J, Smith L.

Centre for Rural Health, University of Tasmania, Australia

**Background:** Implementing community-based mental health programs in rural communities, where the impacts of mental health problems are compounded by isolation, lack of services and concerns about stigma can be particularly challenging. Additional factors, specific to farmers and their families, exacerbate the challenges. These include work, financial and production pressures, as well as cultural factors such as apprehension around help-seeking and a tendency for self-reliance. However, assessment of readiness for change as related to the different community sectors within rural environments may be helpful in enhancing the program's success.

**Aim:** To assess readiness for change and associated variables in four agricultural based rural Tasmanian communities.

**Methods:** A mixed-methods approach using a variety of sampling methods including convenience sampling, snowball sampling, an on-line survey and web links via social media platforms were used to collect data (n = 245). The primary measure employed in the survey component was derived from the Communities Advancing Resilience Toolkit (CART) instrument. Several additional items were included to align the instrument with the research scope including whether the participant was currently involved in farming/agriculture. The survey component was supplemented with qualitative (focus group and interview) data from key local stakeholders (n = 24) at each site. Descriptive statistics were generated from the survey data while the qualitative data were analysed using thematic analysis.

**Results:** Survey data indicated that the four study sites and population sub-groups had different levels of maturity and self-efficacy around key readiness variables such as availability and access to community resources, mental health literacy, community collaboration and service access. Findings from the thematic analysis were consistent with the role of community readiness as a key factor in program success. Perceived barriers to program success included high program staff turnover, lack of clarity around the programs goals, lack of local leadership and challenges with maintaining community interest.

**Conclusion:** Findings were consistent with the view that community readiness is a key factor in the successful delivery of mental health programs in rural areas.

Presenting author: Stuart R Auckland stuart.auckland@utas.edu.au

**S03 - Variation in paediatric emergency presentations across south-western Victoria**

Kloot K, Baker T.

Centre for Rural Emergency Medicine, Deakin University, Australia

**Aim:** Despite some studies examining the common conditions of children presenting to emergency departments in urban areas, very little data exists worldwide on paediatric emergency presentations to rural hospitals. Understanding the diagnoses and severity of patients attending a facility can aid in appropriate resourcing. This study investigates the proportions, conditions, and severity of children attending rural hospitals in south-western Victoria.

**Methods:** Data was extracted from RAHDaR (Rural Acute Hospital Data Register) on all emergency presentations between 1 February 2017 and 31 January 2018 for patients aged less than 18 years. Information included gender, age, presentation and management times, triage category, and diagnosis (collated into Major Diagnostic Block (MDB)) for presentations to the 11 participating hospitals. MDB was not available for one facility. Summary descriptive statistical analysis was performed using IBM SPSS statistical software (Version 22.0, IBM, Armonk, NY, USA) and Microsoft Excel 2013.

**Results:** During the 12 month period there were 13 938 pediatric presentations (23.6%). Significantly different proportions of pediatrics presented across the region (mean 23.7%, range 17.6%–32.2%, χ2 234.6, p < 0.0001): 46% to Level 4 hospitals, 36% to Level 3 and 18% to Level 2. Significantly different proportions of triage category were seen (χ2 2486.581, p < 0.0001). MDB was recorded for 11 207 presentations with the top five being: Digestive system illness; Illness of ear, nose, and throat; Injury, single site, major; Respiratory system illness; and System infection/parasites. These represented 69% of all MDB presentations with a statistically significant difference detected across facilities (χ2 234.6, p < 0.0001).

**Conclusion:** There was a large variation in the presentation numbers at each facility across the region. Presentation severity also showed differences between the hospitals. Despite these variances, over two-thirds of pediatric presentations were related to the same five MDB. These factors have potential impact on the workload at resource-limited facilities.

Presenting author: Associate Professor Timothy Baker tim.baker@deakin.edu.au

**S04 - Discharge planning and access to rehabilitation services after a major farm-related injury**

Beattie J^1^, McLeod C^1^, Murray M^1^, Brumby S^1,2^, Gabbe B^3^.

^*1*^*Deakin University,*^*2*^*National Centre for Farmer Health,*^*3*^*Monash University, Australia*

**Aim:** Farmers are at increased risk of death and over represented in injury statistics compared to other occupations. The majority of research has focused on statistics associated with farm injury morbidity and mortality, yet few studies have explored farmers’ perspectives following major farm injury. To investigate farmers experiences with discharge planning and rehabilitation services after a major farm-related injury.

**Methods:** Participants (18 years and over) who had sustained a major trauma on a farm in Victoria, between 2007 and 2013 were recruited using the Victorian State Trauma Registry (VSTR). Thirty-one participants underwent a qualitative, in-depth semi structured interview. The interviews explored the farmers’ experiences post injury with discharge planning and ambulatory rehabilitation. Interviews were conducted by phone, recorded with a digital voice recorder and transcribed verbatim. Interviews were then coded using NVivo software, subjected to thematic analysis where emergent themes were identified.

**Results:** Participants included a range of employment statuses, including self-employed, full-time farmers, part- time farmers with a supplementary income and agricultural employees. Analysis of participant responses connected to the discharge planning and rehabilitation services identified four major interconnected themes, with associated subthemes: (i) discharge, (ii) communication, (iii) coordination of care, (iv) access to services.

**Conclusion:** There is an opportunity to enhance the rehabilitation process for farmers post discharge. Recommendations include improved communication between tertiary hospital, patient and their regional health service. This would translate to a more coordinated approach to care, access to appropriate rehabilitation and support services and ultimately aid in recovery.

Presenting author: Jessica Beattie j.beattie@deakin.edu.au

**S05 - Child road traffic mortality in Victoria 2001–2012**

Chang SSM, Ozanne-Smith J.

Monash University, Australia

**Background:** Extensive efforts to reduce unintentional injury were enacted in the last three decades of the 20th century. Examination of road traffic injury mortality indicates the extent of fatal, unintentional child injuries.

**Aims:** (1) describe in-depth child road traffic injury (RTI) deaths 2001–2012 in Victoria, Australia (2) identify the potential preventability of the RTI causes.

**Methods:** Fatal Victorian child injury data were extracted from the National Coronial Information System (NCIS) for the 12-year period January 2001 to December 2012. All on-road data was analysed. Data for passenger and pedestrian deaths was examined in depth. Associated factors were determined using univariate and pairwise analysis of factors. Published WHO key prevention strategies, and the recent literature were reviewed, focusing on the identified fatalities among children 0–14 years.

**Results:** For 172 RTI deaths, head injury was the leading medical cause of death (68%). Significantly, the most vulnerable age group for both passengers and pedestrians was 0–4 years. Rural children were over- represented with children aged 0–4 years at greatest risk. Common factors for occupants were loss of control and veering to the incorrect side. For pedestrians the major factors related to rural residence and supervision.

**Conclusion:** This study confirms that RTIs are complex and follow chains of events. Wider implementation of these advanced engineering, education and enforcement strategies may further improve mortality in Victoria. The need for targeted action in rural regions of Victoria is highlighted. The need for a safe systems approach is paramount.

Presenting author: Susan Chang susansoonmee.chang@gmail.com

**S06 -Measure to manage: Using cholinesterase assessment to monitor agrichemical exposure in Victorian farmers**

Brumby SA^1,2^, Russell-Green S^2^, Phillips TJ^1^, Edwards J.^3^

*National Centre for Farmer Health:*^*1*^*Deakin University,*^*2*^*Western District Health Service,*^*3*^*Flinders University, Australia*

**Aim:** As an occupation, farming is not one that regularly monitors blood cholinesterase (ChE) compared to other occupations such as commercial agrichemical handlers and manufacturers regularly exposed to anticholinesterase chemicals. This study aimed to improve farmer awareness of ChE monitoring, validate accurate assessment in-field, and provide farming and agricultural workers with a link between their ChE activity and their agrichemical use.

**Methods:** Cholinesterase (Red Blood cell (AChE), Plasma (PChE) and oxime regenerated PChE) and chemical use data collection took place from April 2016 – March 2017. Health checks and chemical usage questionnaires were conducted pre and post study period. Using an adaptation of a commercially-available cholinesterase test (EQM Test-Mate), PChE measurements were performed in duplicate in the presence and absence of oxime to regenerate PChE in vitro. Individual measures were compared in farmers (n = 64) from four western Victoria locations, at a number of time points, over a calendar year (12 months).

**Results:** Study locations demonstrated different levels of inhibition of PChE consistent with seasonal agricultural practice. The individual repeated measurements were reported to participants and this resulted in improvements in understanding of agricultural chemical risks and improvements in workplace safety practices. The chemical use survey results suggest that gloves are the most commonly used form of PPE used by participants with 39% of farmers surveyed ‘always’ wearing gloves when using or preparing agrichemicals. There was a 66% increase in frequency of respirator usage post-research participation. Pre and post research questionnaires suggest that fewer farmers were using insecticides post involvement in research.

**Conclusion:** Findings suggest regeneration of plasma cholinesterase is a sensitive indicator of exposure to anticholinesterase chemicals. This work has provided proof of application for mobile oxime regeneration of PChE activity in Australian farmers and agrichemical users, highlighting the importance of interactive, participatory models of research to bring about practice change.

Presenting author: Dr Jacqueline L Cotton jacquie.cotton@wdhs.net

**S07 - Blood pressure and its influence on general practitioner visits in rural Ararat**

Gavino A^1,2^, Isaac V^3^, McLachlan C^2^.

^*1*^*East Grampians Health Service,*^*2*^*University of New South Wales,*^*3*^*Flinders University, Australia*

**Aim:** Self-awareness of blood pressure (BP) values may motivate individuals to seek general practitioner (GP) management. GP visits provide opportunities for cardiovascular management; however, an increase in number of visits by repeat patients for BP management has not been previously assessed. Our aim was to determine which factors related to BP are associated with GP visit frequency in a rural community.

**Methods:** Two-hundred and seventy-eight (278) residents of Ararat Rural City responded to a health survey that asked about their sociodemographic profile, medical history, BP awareness and perception, self-rated health (SRH), and frequency of GP visits. Survey booklets were returned and encoded into a research database. Data was analysed using frequency tables and descriptive statistics. Associations were evaluated using Chi-squared test. Multivariate logistic regression analysis was performed to adjust for confounders in the association.

**Results:** Mean age of the cohort was 63.6 ± 12.4 years and 63.3% were women. Hypertension (37.8%) was the most common medical condition. GP visits were made at least once every month (19.1%), every 2–6 months (35.6%), >6 months (11.5%), or only when needed (29.9%). Univariate analyses showed age, education, alcohol consumption, comorbidities, hypertension status, high BP perception and SRH were significantly associated with GP visit frequency. Regression analyses found that having hypertension (OR = 3.4 [95%CI = 1.4–8.3]) and poor SRH (OR = 3.6 [95% CI = 1.5–8.7]) were significantly associated with frequent monthly GP visits, but not perception of having high BP (OR = 0.7 [95%CI = 0.2–2.0]).

**Conclusion:** Based on our rural population sample, we demonstrated that having hypertension and poor perception of health were associated with frequent monthly visits to the GP. The perception of high blood pressure is not a factor that drives additional health-seeking.

Presenting author: Dr Alex Gavino alex.gavino@eghs.net.au

**S08 - Family farm transfers affect family health and individual wellbeing**

Luhrs D.

The Australian Sociological Association, Australia

**Aim:** Intergenerational family-farm transfer is promoted as the most productive and efficient way to ensure a viable and productive farming enterprise across the generations. The research aimed to examine not only the variations of family-farm transfers, but also to examine the effects of intergenerational farm transfers on all family members – parents, successors and non-successors - to highlight related social issues.

**Methods:** The research method involved in-depth, one-on-one, semi-structured interviews with 50 participants from farming families in 11 shires in western Victoria, Australia. Family member participants were sought through advertisements posted on social media, in local newspapers and in the national newspaper *The Weekly Times*. Twenty professionals associated with farming people were also interviewed to augment and triangulate the data obtained from farming family members. They were approached through their business contact numbers and emails. Interviews were transcribed then analysed thematically to determine the main issues arising for family members.

**Results:** Thematic analysis of the interview data revealed a number of issues arising out of intergenerational family-farm transfer decisions and processes that have a direct effect on the relationships between family members and their personal well-being and the functioning of their family. Transfer issues directly affecting personal well-being include the ability of the parental generation to transfer the farm and to retain sufficient income for their retirement; satisfying the career aspirations of the next generation family members; satisfying the desires of non-successors for continued association with the family farm after the transfer to a successor; and actually transferring the farm.

**Conclusion:** Many families and family members, who once enjoyed their shared existences on family farms, struggle with personal issues arising from farm succession. Intergenerational family-farm transfer is a complex social event that requires attention to the emotional well-being of all family members as well as to preparing a farm successor.

Presenting author: Diane Luhrs diane.luhrs@bigpond.com

**S09 - Strengthening Victorian rural women and agriculture: A case-crossover study**

McNamara M^1,2^, Brumby SA^1,2^

*National Centre for Farmer Health:*^*1*^*Deakin University,*^*2*^*Western District Health Service, Victoria, Australia*

**Aim:** In 1998, the Victorian Government supported 20 women to attend the second International Conference on Women in Agriculture, Washington D.C. This gesture followed the rural women's movement and aimed to develop individual capacity to respond rural issues via education and networking. This research evaluates the experience and attendees’ ensuing engagement with rural communities over the past 20 years.

**Methods:** Data were collected from Victorian attendees (20 scholarship and other attendees) via on online survey. Qualitative and quantitative data were collected and each case served as its own control – with participant responses prior to and following the conference period compared and themed. Focus Group discussions gathered qualitative data relevant to themes from the quantitative data. Social network analysis was used to report the connections between people post conference. Ethics approval by Deakin University Health Ethics Advisory Group Health (HEAG_H 147_2017).

**Results:** Thirty attendees completed the online survey, attaining a 97% response rate. Average baseline (1998) age was 43 years (22–59 years), 47% lived on farm and the majority had obtained up to Bachelor level education. Responses showed the experience enhanced knowledge, aptitude, and careers. The majority (90%) of participants claimed the experience increased the profile of women and extended networks (97%). Forty per cent undertook further education, with 63% of participants reporting having attained a Masters level or above by 2017. Ninety-three per cent reported enhanced self-identification as a leader and 93% reported enhanced capacity to respond to rural community issues.

**Conclusion:** Supporting women strengthened skills, leadership capacity and career growth. Increased willingness to engage with rural and agricultural issues is highlighted in involvement in policy and program development. The experience continues to influence attendee actions; the legacy of these women lives on in strategy dialogue, organisational operation and community development

Presenting author: Molly McNamara mcnamaramolly@hotmail.com

**S10 - Exercise and depressive symptoms in older adults: An investigation into psychosocial factors**

Miller KJ^1^, Grace F^1^, Gomez R^1^, McLaren S^1^, Mesagno C^1^, Miloyan B^1^, Yates M^2^.

^*1*^*Federation University Australia,*^*2*^*Deakin University Australia*

**Aim:** Depression is not a normal consequence of biological ageing, yet the prevalence of depression in older adults exceeds 15%. This study tests a theoretical model hypothesising that the relationship between exercise and reduced depressive symptoms is explained by three psychosocial mediators in older adults. This investigation aims to progress the understanding of depression to inform future exercise prescription.

**Methods:** This study employs a cross-sectional analysis of survey data from a sample of 500 community dwelling older Australians aged 65 and over (M = 71.56; range = 65 to 96). Participants reported information on demographics, exercise habits, depressive symptoms, exercise-induced mood, exercise self-efficacy and perceived social support. Bivariate (Pearson) correlations were performed and structural equation modeling (SEM) was subsequently used to test mediation effects in the theoretical model.

**Results:** Current analysis of 331 participants (66.2% of required sample) demonstrates preliminary evidence of a modest, negative relationship between exercise and depressive symptoms. SEM indicates that exercise also has an indirect association with depressive symptoms through exercise-induced mood and social support, but not self-efficacy.

**Conclusion:** To date, the main findings from this study indicate that the exercise–depression relationship is a multifaceted phenomenon. Clinicians and researchers should consider the influence of exercise-induced mood and social support to inform future exercise prescription in older populations.

Presenting author: Kyle J Miller Kylemiller9@outlook.com

**S11 - Rural and regional young people's views on megatrends and their wellbeing**

Moeller-Saxone K, Watt R, McIveen L.

VicHealth, Victoria, Australia

**Aim:** Increasing competition for tertiary education, decreasing job security, globalisation, technology, cultural diversity and over-exposure to the internet; these are some of the issues facing young people. These challenges were identified in 2015 in the Bright Futures Report. VicHealth partnered with National Centre for Farmer Health and YACVIC to explore what this means for young people in rural and regional areas.

**Methods:** Two workshops were conducted with up to 50 young people living in Camperdown and Swan Hill, Victoria, Australia. Participants were asked to comment on the impact of megatrends on the lives of young people living in their area. The workshops were recorded, transcribed and thematically analysed.

**Results:** Young people reported a range of barriers to their wellbeing; they acknowledged that education facilities in regional areas did not always prepare them for work, or that a sufficient range of work opportunities were available locally. A lack of internet access forced them to use smartphones to communicate with friends or use a computer. Few appropriate face-to-face mental health services meant that they were not confident to reach out for help. Facilitators of wellbeing in regional areas included knowing people who could provide jobs; strong support networks developed through exposure to bushfires and other emergencies; and the value of being involved in sporting and other clubs.

**Conclusion:** Megatrends describe major long-term trends affecting society and provide insight into the issues affecting young people in Australia. However, rural and regional young people report that some megatrends are impacting them differently or not at all compared to young people living in urban areas. These are important issues for planners and policymakers who aim to support the wellbeing of young people.

Presenting author: Kristen Moeller-Saxone kmoellersaxone@vichealth.vic.gov.au

**S12 - AgriSafe Australia: Addressing health and safety in farmers and agricultural workers**

Phillips TJ^1,2^, Brumby SA^2^, Hatherell T^1^, Semmens M^1^.

*National Centre for Farmer Health:*^*1*^*Deakin University,*^*2*^*Western District Health Service, Victoria, Australia*

**Aim:** Australian agriculture remains hazardous, reporting no significant reduction in worker fatality rates compared to other industries over the past decade. While other industries share similar hazards, Australian farms are vulnerable – being family owned/operated and reliant on family labour. Poor farmer health and safety practices result in premature death, injury or illness – creating damaging flow-on effects to families and farming businesses.

**Methods:** To address hazards the AgriSafe clinic commenced in 2011. Recruitment of farm men, women and agricultural workers was achieved via referral, hospital presentations, word of mouth, self-referral. The Clinic has three parts: 1. Pre occupational health survey – establish farming/agricultural practice, and occupational exposures. 2. One-on-one health assessment – screening for chronic disease risk factors, psychological distress, occupational health and safety hazards/behaviours. 3. Health and safety coaching/education focusing on prevention of disease, hazard-risk minimisation, recommendations on lifestyle and behaviour changes to improve health, and safety outcomes.

**Results:** The 216 participants who attended an AgriSafe clinic had a mean age 50 years (ranging from 18 to 84). More males (77%) than females (23%) attended; 69.4% of men and women were overweight or obese increasing the potential risk of chronic diseases – heart disease and type 2 diabetes; 54% reported high-risk drinking at least monthly increasing the risk of lifetime and single occasion harm; 51% of quad bike riders never wore a helmet and 52% reported hearing loss, with 91% wearing hearing protection at least sometimes and 37.5% always wearing hearing protection.

**Conclusion:** AgriSafe Clinics have identified serious and risky practices both in safety and health behaviours through identification of poor health, connecting agricultural hazards to farmers and their practices. Follow-up evaluation is currently underway to assess change of behaviours, reduction of risks and action of referrals.

Presenting author: Tam J Phillips tam.phillips@wdhs.net

**S13 - Cardiac telerehabilitation combines near-universal accessibility with expert oversight: Protocol for the SCRAM RCT**

Rawstorn JC^1^, Ball K^1^, Oldenburg B^2^, Chow C^3^, McNaughton S^1^, Lamb K^4^, Goa L^5^, Moodie M^5^, Amerena J^6^, Nadurata V^7^, Neil C^8^, Maddison R^1^.

^*1*^*Institute for Physical Activity and Nutrition, Deakin University,*^*2*^*Centre for Health Equity, University of Melbourne,*^*3*^*Sydney Medical School, University of Sydney,*^*4*^*Murdoch Children's Research Institute,*^*5*^*Centre for Population Health Research, Deakin University,*^*6*^*Barwon Health,*^*7*^*Bendigo Health,*^*8*^*Western Health Service, AU.*

**Aim:** Cardiac rehabilitation (CR) saves lives and improves wellbeing but key accessibility barriers stop many people from participating – especially in regional areas where access to face-to-face rehabilitation is lowest. Our cutting-edge telerehabilitation intervention (SCRAM) remotely connects people with rehabilitation specialists to receive evidence-based rehabilitation support regardless of their location. Our NHMRC-funded trial will evaluate the effects and costs of SCRAM.

**Methods:** A multi-centre, single-blind, parallel arm randomised controlled trial will assess the effects and costs of SCRAM among 220 people with coronary heart disease in the Barwon, Western and Bendigo Health catchment regions. All participants will retain access to usual care CR and half will also receive SCRAM—a 24 week bi-phasic program of exercise training and behaviour change support delivered via a bespoke telerehabilitation platform. Outcomes include exercise capacity, medical and lifestyle risk factors, program delivery costs, and cost-effectiveness. A mixed methods process evaluation will assess user experiences.

**Results:** Recruitment began in August 2018.

**Conclusion:** SCRAM overcomes key accessibility barriers while retaining a high level of oversight and support from rehabilitation specialists. If proven cost-effective, this world-leading delivery model could greatly increase the impact of CR by reaching the many people in regional areas who have limited access to traditional face-to- face services.

Presenting author: Dr Jonathan C Rawstorn jonathan.rawstorn@deakin.edu.au

**S14 - Post-arthroplasty review: Can we replace outpatient appointments with a remote review model?**

Reynolds B^1^, Maister N^2^, Gill S^3^, Waring S^2^, Schoch P^1^, Beattie S^3^, Thomson A^2^, Page RS^3^.

^*1*^*Physiotherapy Department, Barwon Health,*^*2*^*Orthopaedic Department, Barwon Health,*^*3*^*Barwon Centre for Orthopaedic Research and Education, St John of God Geelong Hospital and Barwon Health, Victoria, Australia*

**Aim:** The number of hip and knee replacements will double over the next decade. In public hospitals, regular post-surgical outpatient review has commonly occurred for the duration of a patient's life. However, there is limited evidence regarding the utility of these reviews for identifying complications, particularly in regional and rural locations. We investigated when and where post-surgical complications were identified.

**Methods:** The medical records of all patients requiring re-operation for complications following primary hip replacement (n = 48, 2004 to 2015) or primary knee replacement (n = 50, 1998 to 2015) at University Hospital Geelong were inspected. Data were extracted by one of four investigators using a standardised electronic data extraction tool. Variables of interest included the health setting where the complication was initially identified, how long following surgery that the complication was identified and whether the complication was symptomatic.

**Results:** Routine post-surgical appointments identified 15 (15.3%) complications requiring re-operation; all were identified in the first ear post-surgery. For each complication identified in the first year post-surgery,

approximately 1000 outpatient appointments were required. After the first year, all complications were identified in Emergency Departments (n = 30, 30.6%), General Practice (n = 24, 24.5%) or non-routine orthopaedic outpatient appointments (n = 19, 19.4%). All patients with complications reported symptoms.

**Conclusion:** Routine post-surgical outpatient appointments were an inefficient mechanism for identifying complications requiring re-operation more than one year following surgery. We subsequently redesigned our services to include a remote questionnaire-based model, which can be used by other regional and rural health services to reduce the burden on patients and outpatient services.

Presenting author: Bill Reynolds bill.reynolds@barwonhealth.org.au

**S15- Twenty-minute rounding: Is this the solution to reducing falls and factures?**

Roberts BJ^1^, Garda A^1^, Holloway K^2^, Pretorius T^1^, Kennedy AJ^2^, Saligari H^1^, Beks H^2^, Armstrong K^1^.

^*1*^*Western District Health Service,*^*2*^*Deakin University*

**Aim:** This research explored the impact of ‘staff rounding’ on the incidence of falls and falls-related injuries among high falls-risk residents within aged care. Residents were randomly allocated to the intervention or control group. Rounding data was recorded in the residents’ notes every 20 minutes for six months, and identified resident risk reduction of falls and falls-related injuries.

**Methods:** Continuous variables were compared between the control and intervention groups using t-tests for parametric data and the Mann- Whitney test for non-parametric data. Categorical variables were compared using Chi-square tests. Differences between the two groups for incidence of falls were compared using t-tests. Additional investigation was conducted to determine the impact of other factors (age, sex, medication use, site, PAS scores, FRAT scores, etc.) on falling using logistic regression techniques.

**Results:** There was a trend (p1 = 0.056) indicating a reduction in falls during the trial, compared to the six months prior. In the preceding six months, there were an average (±SD2) of 60.4 ± 35.7 falls, compared to an average of 53.4 ± 37.4 during the trial. This corresponded to a difference of 7.0 ± 5.9 falls across the two time periods. One site was an outlier in this analysis. Repeating the analysis with this site excluded, the above association of a reduction in falls is significant, with an average of 48.8 ± 28.2 falls in the previous six months and 39.8 ± 25.0 falls during the trial (difference 9.0 ± 4.4 falls, p = 0.026).

**Conclusion:** A major finding of the research was that increased observation among the intervention group led to zero ISR 1 or 2 falls, which indicates a significant positive impact on the quality of life for aged care residents. The study provided significant challenges and lessons, which will guide further research.

Presenting author: Bronwyn Roberts bronwyn.roberts@wdhs.net

**S16 - Supporting farmer mental health through resilience interventions: Measuring, monitoring and building resilience**

Schirmer J, Corocher L, Hanigan IC.

Sydney Medical School, University of Sydney

**Aim:** The NSW Department of Primary Industries Rural Resilience Program (RRP) works to build resilience in farming communities, through activities such as strengthening farmer networks, exchanging information, and delivering initiatives that build personal and business resilience skills. In this study the University of Canberra worked with RRP to develop measures that enable better monitoring of resilience, and identification of intervention needs.

**Methods:** This project first drew on existing literature to propose how best to measure and monitor different dimensions of farmer resilience. Measures were recommended for identifying emerging resilience challenges and changes in the short-term and longer-term. The RRP is currently testing short-term measures that provide rapid assessment of resilience challenges. Regional Wellbeing Survey data were used to construct longer-term measures and test their useability and practicality for identifying resilience to climate- related challenges such as drought, drawing on a sample of 1400 NSW farmers who completed the survey in 2015.

**Results:** Findings showed psychological distress and wellbeing differed by age and farm enterprise, suggesting intervention based on life stage and farm type is as important as targeting interventions based on geographic location. Interventions can build resilience effectively if targeted at building and maintain (i) self-efficacy (skills, knowledge, and capability) and (ii) building supportive social networks. While supporting farmer health and farm finances in difficult times is also important, the study findings suggest that building self-efficacy and social resources provides resources farmers can draw on to help maintain other resilience resources in difficult times, including farm finances and health.

**Conclusion:** Early intervention is important to reduce impacts of events such as drought that can pose a risk to farmer health. This study provides a practical approach to measuring and monitoring resilience that can be used to identify emerging challenges as well as monitor success of interventions.

Presenting author: Jacki Schirmer jacki.schirmer@canberra.edu.au

**S17 - Data analytics and community based screening for undiagnosed diabetes in a rural setting**

Stranieri A^1^, Jelinek H^2^.

^*1*^*Federation University Australia,*^*2*^*Charles Sturt University*

**Aim:** Chronic diseases including diabetes, cardiovascular disease, obesity and metabolic syndrome conditions are increasing globally at alarming rates. A substantial proportion of chronic diseases remain undiagnosed. Although community screening for undiagnosed chronic conditions meets the 10 criteria advanced to assess the effectiveness of screening programs, these programs are not commonplace.

**Methods:** A community based screening program has been run by student nurses on placement around Albury over the past decade. Data on over 300 variables from more than 2500 clinic attendances has been collected representing demographic indicators, glycated haemoglobin (HbA1c), cholesterol, inflammatory and oxidative stress markers, Body-Mass Index (BMI), peripheral vascular function, ECG and many other test results. Data analytics with machine learning on this unique dataset has been performed to discover co- markers that, together with glycated haemoglobin (HbA1c) can identify undiagnosed diabetes with fewer false positives than HbA1c alone.

**Results:** Optimal thresholds of Interleukin-6 (IL-6), urine 8-hydroxy- deguanosine (8-OHdG), Heart rate variability sample entropy and Atherogenic Index of Plasma were discovered that, when combined with thresholds on HbA1c, predicted diabetes with specificity above 88% and sensitivity above 81%. Each one of these markers used with HbA1c reduces the false positive diabetes predictions that arise from HbA1c alone.

**Conclusion:** Community based screening for chronic conditions run by student nurses can be shown to be run cost-effectively. The dataset generated from community based screening can be used with data analytics algorithms to discover markers for undiagnosed chronic conditions.

Presenting author: Associate Professor Andrew Stranieri a.stranieri@federation.edu.au

**S18 - Family burden and social support in mental illness**

Thomas JT^1^, Petrakis M^2^, Sheehan P^2^.

^*1*^*South West Healthcare Mental Health Services,*^*2*^*Monash University*

**Aim:** The de-institutionalisation movement that began in 1970 s of taking the mentally ill persons out of mental hospitals into the community has put enormous stress on families and the task of caring for them falls largely on family members. The present study was an attempt to assess family burden and social support perceived by both consumers and their caregivers.

**Methods:** The study was carried out at the Institute of Mental Health (IMH), Hyderabad, India with a capacity of 600 in-patient beds. The study adopted a quantitative descriptive study design: 54 persons affected with schizophrenia and 58 with mood disorders, and one of their key relatives were, selected purposively and consecutively meeting certain inclusion and exclusion criteria. The global assessment of functioning, family burden, and social support perceived by both consumers and caregivers were assessed by using standardised scales. The statistical analysis was carried out by using SPSS (version 23) package.

**Results:** The persons affected with schizophrenia were found to be more severely ill (p < 0.001) with a longer duration of illness (p < 0.001) and perceived less social support (p < 0.001) as compared to the persons affected with mood disorders. The caregivers’ perceived social support was similar in both diagnostic groups. Family burden was significantly (p < 0.001) higher in the schizophrenia group. Interestingly the social support perceived by the caregivers was minimal and similar in both the diagnostic groups. Some significant correlations have been found among socio-demographic and clinical characteristics, and the key study variables.

**Conclusion:** The study findings revealed that the pattern of caregiver burden was similar in both schizophrenia and mood disorder groups. The caregivers in both the groups did not receive any support outside the family. These findings call for interventions aimed at enhancing the social support for both consumers and their caregivers.

Presenting author: Dr Josy K Thomas jkthomas@swh.net.au

**S19 - High-intensity interval training in patients with cancer: A systematic review and meta-analysis**

Wallen M^1^, Wong Shee A^2,3^, Evans L^3^, Brown S^3^, Rawstorn J^2^, Harvey J^1^, Grace F^1^.

^*1*^*Federation University Australia,*^*2*^*Deakin University,*^*3*^*Ballarat Health Services*

**Aim:** Higher cardiorespiratory fitness (CRF) is associated with reduced cancer-related mortality, recurrence and improved treatment outcomes. High-intensity interval training (HIIT) could offer a more efficient method to improve CRF than traditional moderate intensity continuous training (MICT). Our systematic review and meta- analysis compared the impact of HIIT versus usual care (UC) and/or MICT on CRF in cancer patients and survivors.

**Methods:** We systematically reviewed trials of HIIT versus UC and/or MICT in cancer patients and survivors. PubMed, Medline and Web of Science databases were searched (inception–11/2017) for all studies comparing HIIT to UC and/or MICT in cancer patients and survivors. Inclusion criteria were: participants aged ≥18 years; pre- and postintervention evaluation of peak oxygen uptake (VO2 peak); intervention duration ≥3 weeks; HIIT was predominantly aerobic-based exercise, interspersed by active or passive recovery periods; UC and/or MICT groups.

**Results:** Six randomised controlled trials (n = 346) and one quasi-experimental trial (n = 35) were analysed. Three studies examined HIIT in the preoperative period for patients with colorectal-liver metastases, lung and rectal cancers. Four studies implemented HIIT after treatment, involving patients diagnosed with colorectal, breast, testicular, ovarian or vaginal cancer, non-invasive urothelial carcinoma or non- Hodgkin's lymphoma. HIIT protocols varied widely (i.e. interval/rest duration, exercise intensity). HIIT significantly increased VO2 peak by 4.28 mL·kg-1·min-1 compared to UC. Improvements were comparable for patients undertaking HIIT before (3.98 mL·kg-1·min-1) and after treatment (3.72 mL·kg-1·min-1). Compared to MICT, HIIT significantly improved VO2 peak by 3.80 mL·kg-1·min-1. No serious adverse events were reported.

**Conclusion:** HIIT appears safe and significantly improves VO2 peak in cancer patients and survivors when compared to UC and MICT. Similar improvements in VO2peak were demonstrated in patients undertaking HIIT before or after treatment.

Presenting author: Dr Matthew P Wallen m.wallen@federation.edu.au

**S20 - Engaging in antenatal care: An interview study of teenage women**

Frawley N^1^, Wong Shee A^1,2^, Robertson C^1^, McKenzie A^1^, Lodge J^1^, Versace V^2^, Shotton A^3^, Sturmfels K^4^, Nagle C^5^.

^*1*^*Ballarat Health Services,*^*2*^*Deakin University,*^*3*^*Maryborough District Health Service,*^*4*^*East Grampians Health Service,*^*5*^*James Cook University*

**Aim:** Teenage pregnancy is associated with a large societal and personal burden, worldwide. The rate of teenage pregnancy at Ballarat Health Services (14.2/1000 births) was higher than the Victorian average of 10/1000 births. Attendance for pregnancy care is associated with improved outcomes. This study aimed to explore the barriers and facilitators to engagement with pregnancy care providers experienced by teenage women.

**Methods:** Semi-structured interviews were conducted with women who were pregnant aged ≤ 19 years from Ballarat, Ararat and Maryborough health services between February and June 2017. Interviews were audio taped and professionally transcribed. Data was analysed by two researchers independently using thematic analysis guided by Braun and Clarke's approach.

**Results:** Transcripts of interviews with 16 women were analysed and four themes identified: Valuing pregnancy care, Interactions with maternity service, Women-centred care, and Support systems. Young women were motivated to attend to ensure the wellbeing of their baby and lack of engagement occurred when the importance of care was not understood. Flexibility of appointments and a central location was important; most participants were highly reliant on others for transport. Continuity of care and carer were valued and the interpersonal skills of staff strongly influenced engagement. Many women had fractured families and pregnancy led to a contraction of their social world.

**Conclusion:** This study has provided an understanding of the motivating reasons and external influences affecting engagement in antenatal care for teenage women in rural and regional areas. These findings have informed the development of best practice guideline for teenage pregnancy care.

Presenting author: Associate Professor Anna Wong Shee Anna.WongShee@bhs.org.au

Western Alliance Health Research Ltd is a Registered Health Promotion Charity, trading as Western Alliance Academic Health Science Centre and Network https://www.westernalliance.org.au/

